# Hydrodissection and hydrodilatation for joint pain: a scoping review

**DOI:** 10.3389/fpain.2026.1851291

**Published:** 2026-07-23

**Authors:** Benjamin Leahy, Harry McGrath, Dominic Harmon

**Affiliations:** Department of Anaesthesia and Pain Medicine, University Hospital Limerick, Limerick, Ireland

**Keywords:** adhesive capsulitis, hydrodissection, joint pain, musculoskeletal pain, systematic review, ultrasound-guided injection

## Abstract

**Objective:**

To evaluate the current evidence regarding the effectiveness and safety of ultrasound-guided hydrodissection (US-HD) in the management of joint-related pain.

**Methods:**

A systematic search of PubMed, Embase, Cochrane Library, and Google Scholar was conducted for studies published in the past 15 years. Eligible studies included randomised controlled trials (RCTs), cohort studies, and case series investigating hydrodissection for joint pain (e.g., shoulder, hip, knee, sacroiliac joint). Outcomes included pain reduction, functional improvement, and complication rates. Data extraction and qualitative synthesis were performed due to heterogeneity in study design and outcome measures.

**Results:**

A total of 138 studies were identified, of which 21 met inclusion criteria. Evidence suggests that US-HD may provide significant pain relief and functional improvement in conditions such as adhesive capsulitis, greater trochanteric pain syndrome, and knee osteoarthritis. Studies consistently reported low complication rates. However, heterogeneity in technique, injectate composition, and outcome measures was noted.

**Conclusion:**

Ultrasound-guided hydrodissection appears to be a safe and promising intervention for joint-related pain. While early evidence is encouraging, larger high-quality trials are required to establish standardised protocols and confirm long-term efficacy.

## 1 Introduction

Joint-region pain is increasingly understood as a multifactorial phenomenon extending beyond isolated intra-articular pathology, supporting broader treatment approaches such as hydrodissection. Intra-articular contributors—including osteoarthritis, synovitis, labral or meniscal injury, and effusion—are common but demonstrate a poor correlation between structural abnormalities and pain severity, with similar imaging findings frequently observed in asymptomatic individuals. Peri-articular structures play a significant role, with tendinopathy, bursitis, ligamentous dysfunction, and myofascial pain often acting as primary pain generators; notably, tendinopathy along a reactive–degenerative continuum is a key driver of persistent symptoms and responds more effectively to load-based and targeted interventions than passive treatments. Neural mechanisms further contribute, including peripheral sensitisation, nerve entrapment, radicular pain, and central sensitisation, the latter characterised by disproportionate pain, allodynia, and symptom spread independent of local tissue damage. Systemic inflammatory conditions, such as rheumatoid arthritis, seronegative spondyloarthropathies, and crystal arthropathies, contribute via cytokine-mediated pathways that amplify both local and central pain processes. Additionally, psychosocial factors—including fear avoidance, catastrophising, sleep disturbance, and mood disorders—are strong predictors of pain chronicity, disability, and treatment outcomes. Collectively, this multidimensional model underscores the limitations of purely intra-articular interventions and supports more comprehensive, mechanism-based management strategies.

Hydrodissection is a minimally invasive interventional technique that involves the targeted injection of fluid to separate tissue planes under imaging guidance. Initially described in the context of peripheral nerve entrapment syndromes, its application has expanded into musculoskeletal medicine, particularly in the management of joint and periarticular pain. The theoretical basis of hydrodissection in joint pathology lies in its ability to disrupt adhesions, restore normal tissue gliding, and reduce mechanical and inflammatory contributors to pain.

Joint pain is a highly prevalent clinical problem, contributing significantly to disability and reduced quality of life worldwide. Conditions such as adhesive capsulitis of the shoulder, osteoarthritis of the knee, and periarticular syndromes including greater trochanteric pain syndrome are commonly encountered in both primary care and specialist settings. Traditional management strategies include pharmacological therapy, physiotherapy, corticosteroid injections, and surgical intervention in refractory cases. However, these approaches may provide limited or temporary relief, and are often associated with side effects or procedural risks.

Corticosteroid injections are currently the predominant treatment strategy. Corticosteroid injection, while effective for short-term symptom relief, has well-documented deleterious effects on tendon structure and function, which provides a rationale for alternative approaches such as hydrodissection. Steroids impair tendon integrity by reducing type I collagen synthesis and increasing collagen degradation, resulting in a weaker and more disorganized extracellular matrix. They also inhibit tenocyte proliferation and promote apoptosis, thereby disrupting normal tissue repair and regeneration. These cellular and structural changes translate into reduced mechanical properties, with tendons demonstrating decreased stiffness and tensile strength following exposure. Clinically, this is associated with an increased risk of tendon rupture—most notably in the Achilles, rotator cuff, and patellar tendons—particularly with repeated or intratendinous injections. Furthermore, corticosteroids suppress the early inflammatory phase of healing, leading to delayed recovery and inferior repair tissue. Prolonged exposure may also accelerate matrix degeneration, contributing to progression of tendinopathy and transformation from a reactive to a degenerative state.

Hydrodissection offers a potential alternative that combines both mechanical and pharmacological mechanisms. By injecting fluid into the pericapsular or periarticular space, the technique aims to physically separate fibrotic or inflamed tissues, reduce nociceptive signalling, and improve joint mobility. The addition of agents such as dextrose, local anaesthetic, corticosteroids, or platelet-rich plasma (PRP) may further enhance therapeutic outcomes through anti-inflammatory or regenerative effects.

Ultrasound guidance has played a critical role in the evolution of hydrodissection, enabling real-time visualization of anatomical structures, accurate needle placement, and confirmation of injectate spread. This has improved both the safety and efficacy of the technique, making it increasingly accessible in outpatient settings.

Despite growing clinical interest, the evidence base for hydrodissection in joint pain remains fragmented. Studies vary widely in terms of target pathology, procedural technique, injectate composition, and outcome measures. This heterogeneity poses challenges for interpretation and limits the ability to draw firm conclusions regarding its clinical utility.

This scoping review aims to synthesise the current literature on ultrasound-guided hydrodissection for joint-related pain, with a focus on effectiveness, safety, and technical considerations. By consolidating available evidence, we aim to provide a clearer understanding of its role within contemporary pain management strategies.

## 2 Objectives

- To evaluate the effectiveness of ultrasound-guided hydrodissection in joint-related pain conditions
- To assess safety outcomes and complication rates
- To summarise current procedural techniques and evaluate current procedural characteristics of US-HD in joint-related pain

## 3 Methods

### 3.1 Search strategy

A structured literature search was conducted using PubMed, Embase, Cochrane Library, and Google Scholar. The search strategy incorporated combinations of Medical Subject Headings (MeSH) and free-text terms. Key search terms included:

- “hydrodissection” AND “joint pain”
- “ultrasound-guided hydrodissection” AND (“shoulder” OR “hip” OR “knee” OR “sacroiliac”)
- “adhesive capsulitis” OR “capsular distension” AND “hydrodissection”

Boolean operators were used to refine search results. Searches were limited to studies published between January 2010 and March 2025 and restricted to English-language publications. One PubMed search string has been included in Supplementary Material 1 section.

### 3.2 Study selection

Titles and abstracts were screened independently to identify potentially eligible studies. Fulltext articles were then reviewed against predefined inclusion and exclusion criteria. Disagreements were resolved through discussion.

### 3.3 Inclusion criteria

- Adult patients (≥18 years) with joint-related or periarticular pain
- Intervention involving ultrasound-guided hydrodissection
- Reported outcomes including pain scores, functional measures, or safety data
- Study designs including RCTs, prospective or retrospective cohort studies, and case series

### 3.4 Exclusion criteria

- Non-ultrasound-guided procedures
- Intra-articular injections without a hydrodissection component
- Animal or paediatric studies
- Narrative reviews, editorials, and duplicate publications

### 3.5 Data extraction and synthesis

Data extracted included study design, sample size, patient population, target joint, procedural technique, injectate type and volume, outcomes, and complications. Due to methodological heterogeneity, a meta-analysis was not feasible, and a qualitative synthesis was performed.

### 3.6 PRISMA framework

**Figure 1:**
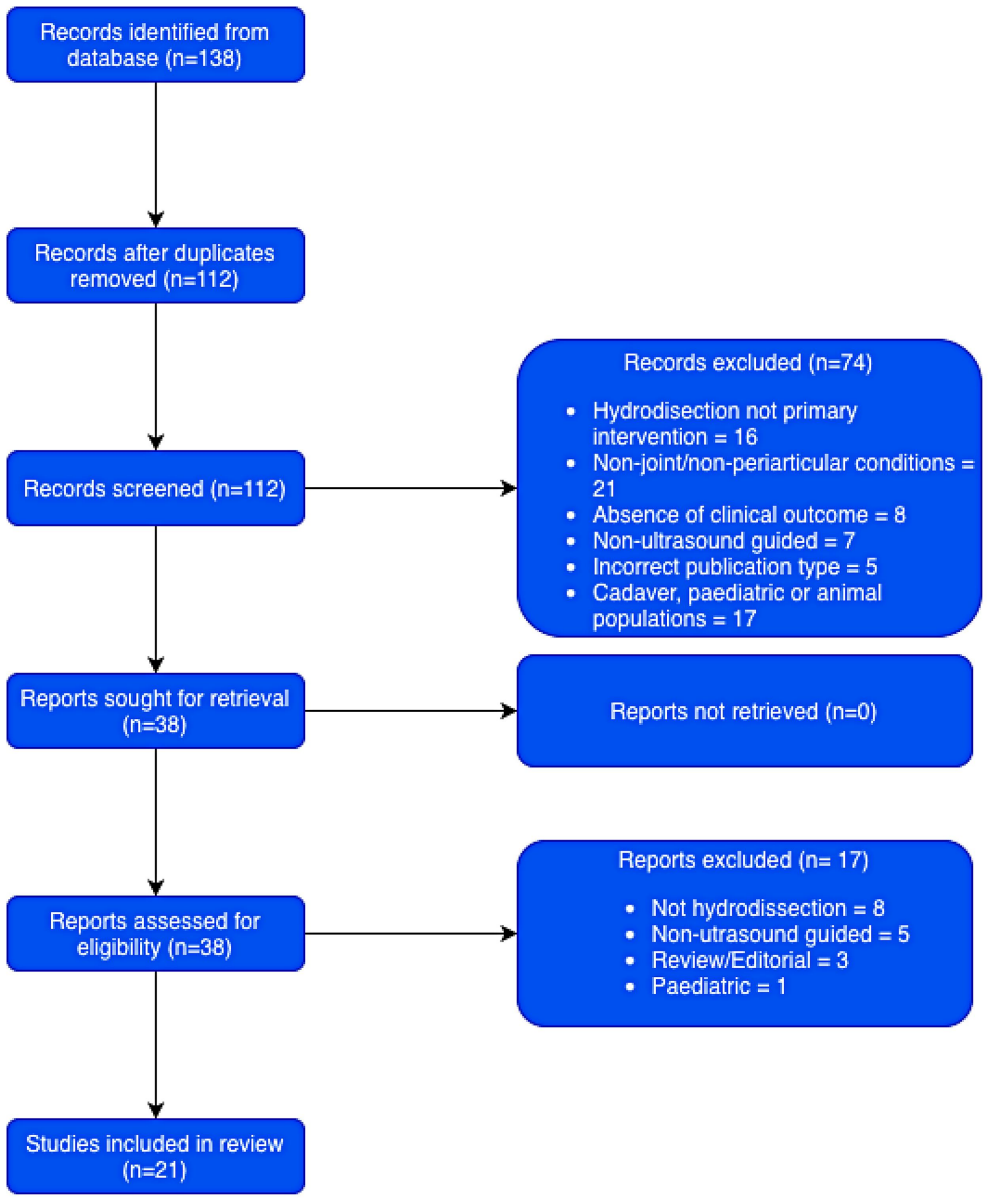
PRISMA Flow Diagram.

The study selection process followed PRISMA (Preferred Reporting Items for Systematic Reviews and Meta-Analyses) guidelines. A flow diagram illustrating study identification, screening, eligibility, and inclusion is described (Figure 1).

## 4 Results

### 4.1 Study characteristics

A total of 138 records were identified. Following screening and eligibility assessment, 21 studies were included.

These comprised:

- 3 randomised controlled trials
- 1 pilot randomised trial
- 3 cohort studies
- 1 retrospective study
- 9 case reports
- 3 case series
- 1 case report and review

Above is a table detailing the risk of bias assessment for the studies included.

**Figure 2:**
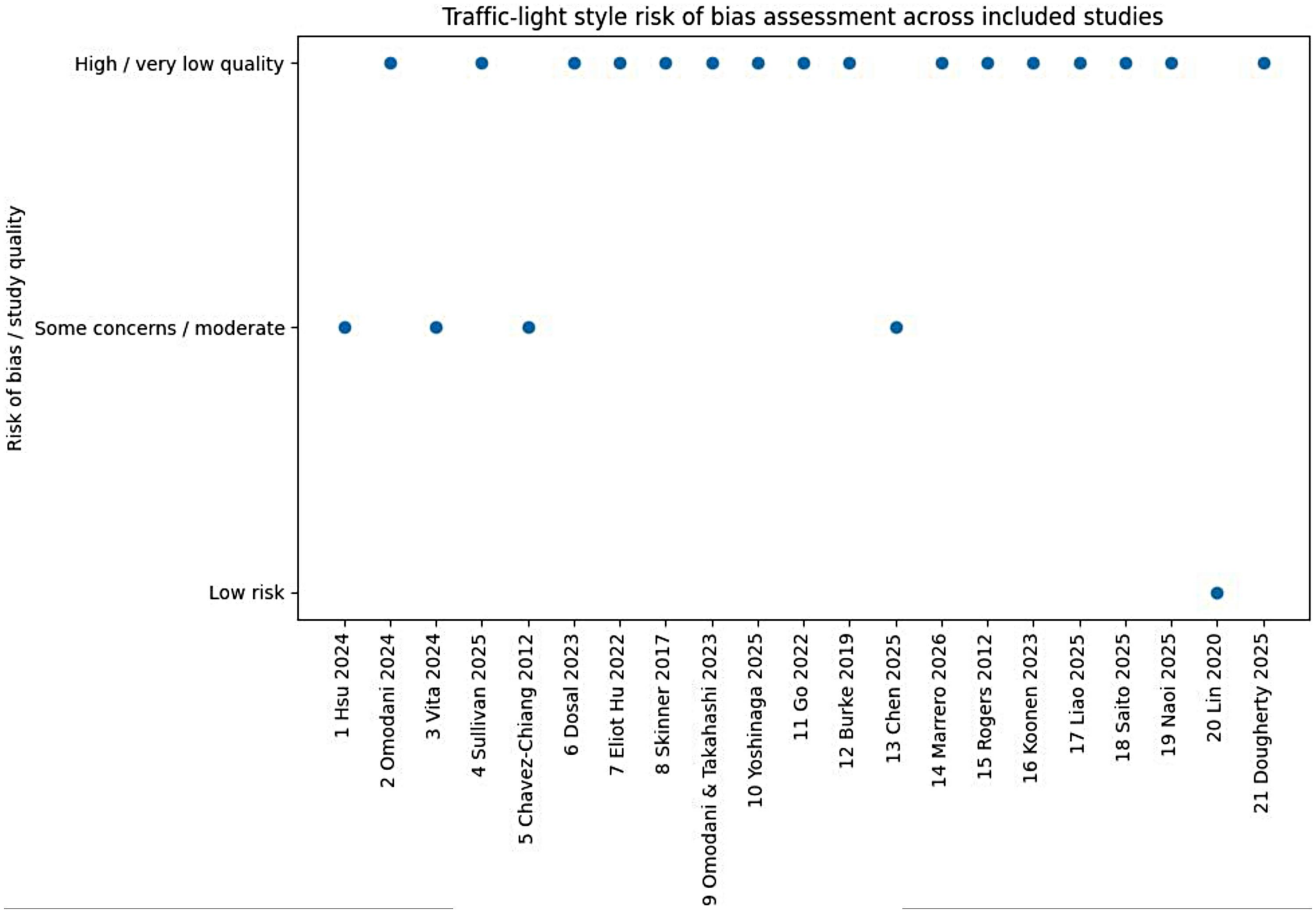
Traffic-light summary of the overall methodological quality and risk of bias of included studies. Each point represents the overall assessment for an individual study following appraisal with the RoB 2, Newcastle–Ottawa Scale, or Joanna Briggs Institute critical appraisal tools, as appropriate.

The overall evidence base is therefore low quality and at high risk of bias (Figure 2, Table 1).

**Table 1:** Assessment of study quality.

No.	Study (Author, Year)	Study design	Appraisal tool	Quality/RoB assessment
1	Hsu et al. 2024 (1)	RCT	RoB2	Some concerns–High risk
2	Omodani 2024 (2)	Case report	JBI	Very low quality
3	Vita et al. 2024 (3)	Prospective study (no control)	NOS	Moderate quality (5/9)
4	Sullivan et al. 2025 (4)	Case report	JBI	Very low quality
5	Chavez-Chiang et al. 2012 (5)	Cohort study	NOS	Good quality (7/9)
6	Dosal et al. 2023 (6)	Case series	JBI	Very low quality
7	Eliot Hu et al. 2022 (7)	Case report	JBI	Very low quality
8	Skinner 2017 (8)	Narrative/case-based	Not applicable	Very low quality
9	Omodani & Takahashi 2023 (9)	Case report	JBI	Very low quality
10	Yoshinaga et al. 2025 (10)	Case report	JBI	Very low quality
11	Go et al. 2022 (11)	Case report	JBI	Very low quality
12	Burke et al. 2019 (12)	Case series	JBI	Very low quality
13	Chen et al. 2025 (13)	Retrospective cohort	NOS	Moderate quality (5/9)
14	Marrero Borrero et al. 2026 (14)	Case report	JBI	Very low quality
15	Rogers et al. 2012 (15)	Case report	JBI	Very low quality
16	Koonen et al. 2023 (16)	Pilot RCT	RoB 2	High risk
17	Liao et al. 2025 (17)	Prospective study (no control)	NOS (limited applicability)	Low quality
18	Saito et al. 2025 (18)	Case series (*n* = 2)	JBI	Very low quality
19	Naoi et al. 2025 (19)	Case report	JBI	Very low quality
20	Lin et al. 2020 (20)	Double-blind RCT	RoB 2	Low risk
21	Dougherty et al. 2025 (21)	Case report	JBI	Very low quality

### 4.2 Effectiveness

#### 4.2.1 Adhesive capsulitis (frozen shoulder)

Adhesive capsulitis was the most extensively studied indication for hydrodissection. Several randomised controlled trials demonstrated significant improvements in pain scores and shoulder range of motion following capsular hydrodissection. These benefits were often observed within weeks of the procedure and were sustained for up to six months in some studies.

Hydrodissection in this context typically involved capsular distension with relatively large volumes (20–40 mL) of saline, often combined with corticosteroid and local anaesthetic. The mechanical expansion of the joint capsule is thought to disrupt adhesions and improve capsular compliance.

Comparative studies suggested that hydrodissection may provide superior short-term functional improvement compared to intra-articular steroid injection alone. However, longterm outcomes were less consistently reported.

#### 4.2.2 Knee osteoarthritis

Evidence for hydrodissection in knee osteoarthritis is more limited but suggests potential benefit. Studies focusing on periarticular hydrodissection, rather than purely intra-articular injection, reported modest reductions in pain and improvements in function. Target structures included the suprapatellar pouch, medial retinaculum, and peri-tendinous tissues.

The mechanism in osteoarthritis is likely multifactorial, involving reduction of soft tissue tension, improved synovial fluid distribution, and possible neuromodulation.

#### 4.2.3 Greater trochanteric pain syndrome (GTPS)

Several case series reported positive outcomes following hydrodissection of the gluteal tendons and iliotibial band in patients with GTPS. Patients experienced reductions in lateral hip pain and improvements in mobility. Although encouraging, these findings are limited by the absence of control groups and small sample sizes.

#### 4.2.4 Other joint applications

Emerging evidence suggests potential roles for hydrodissection in sacroiliac joint dysfunction and hip impingement syndromes. However, these applications remain under-investigated, and current data are insufficient to draw firm conclusions.

Overall, hydrodissection appears most effective in conditions characterised by fibrosis, adhesions, or restricted tissue mobility.

#### 4.2.5 Overall

Clinical outcomes across the included studies consistently demonstrated meaningful reductions in pain following ultrasound-guided hydrodissection. Pain was most commonly measured using the Visual Analogue Scale (VAS) or Numerical Rating Scale (NRS/NPRS). Among studies reporting quantitative outcomes, pain reductions ranged from approximately 3 to 6 points on a 10-point scale. For example, Hsu et al. reported a reduction in VAS scores from 3.7 to 1.4 in patients with myofascial pain syndrome, while Vita et al. demonstrated improvement from 7.65 to 1.65 in De Quervain's tenosynovitis. Sullivan et al. observed a decrease from 8 to 3 in patients with adhesive capsulitis and osteoarthritis, and Liao et al. reported improvement from 7 to 2. Similar benefits were observed in neuropathic, tendinopathic, and post-surgical pain conditions.

Functional outcomes were reported using a variety of validated instruments, including the Disabilities of the Arm, Shoulder and Hand (DASH) score, Shoulder and Neck Disability Index, and condition-specific functional assessments. Across studies, improvements were observed in upper and lower limb function, return to sport, walking tolerance, grip strength, and activities of daily living.

A table with a summary of this data is available in Supplementary Material 3 and a table containing information about the four main pathologies are available in Supplementary Material 4.

### 4.3 Safety and complications

Across all included studies, ultrasound-guided hydrodissection demonstrated a favourable safety profile. No major complications such as infection, neurovascular injury, or permanent disability were reported.

Minor and transient adverse effects included:

- Localised pain or discomfort at the injection site
- Temporary stiffness or swelling
- Mild vasovagal reactions

The use of ultrasound guidance was consistently emphasised as a key factor in minimising procedural risk. Real-time visualisation allows avoidance of critical structures and ensures accurate delivery of injectate.

The outpatient nature of the procedure, absence of general anaesthesia, and rapid recovery further contribute to its safety and acceptability.

### 4.4 Technique and injectates

Significant variability was observed in procedural techniques across studies.

#### 4.4.1 Needle approach

Both in-plane and out-of-plane approaches were described. The in-plane technique allows continuous visualisation of the needle shaft and tip, improving precision and safety.

#### 4.4.2 Target structures

- Shoulder: Glenohumeral capsule, rotator interval
- Knee: Periarticular soft tissues, suprapatellar pouch
- Hip: Gluteal tendons, iliotibial band

#### 4.4.3 Injectates

Commonly used injectates included:

- Normal saline
- 5% dextrose solution
- Local anaesthetics (e.g., lidocaine)
- Corticosteroids
- Platelet-rich plasma (PRP)

Dextrose has been proposed to have neuromodulatory effects, while PRP may contribute to tissue healing.

#### 4.4.4 Volume of injection

Volumes varied widely, ranging from 5 mL in small peri-tendinous spaces to over 40 mL in capsular distension procedures.

Despite these differences, core principles remained consistent: precise needle placement, slow injectate delivery, and visualization of tissue separation.

## 5 Discussion

This scoping review highlights ultrasound-guided hydrodissection as a promising and evolving intervention in the management of joint-related pain. The technique offers a unique combination of mechanical and pharmacological effects, distinguishing it from conventional injection therapies.

The strongest evidence exists for adhesive capsulitis, where hydrodissection appears to improve both pain and function. The mechanical disruption of capsular adhesions is likely a key mechanism, supported by the consistent use of high-volume injectates in these studies.

In knee osteoarthritis and periarticular syndromes, the benefits of hydrodissection may be more modest but still clinically meaningful. The ability to target soft tissue structures rather than solely intra-articular pathology represents an important advantage. Treating both pathologies of intraarticular pathology and soft tissue structures simultaneously is a likely advantage. These concurrent pathologies are common in joint pain.

Safety outcomes are consistently favourable, reinforcing the role of ultrasound guidance in modern interventional practice. The low complication rate compares favourably with other invasive procedures, including surgical interventions.

However, several limitations must be considered. The heterogeneity of study designs, techniques, and outcome measures limits comparability. Many studies are small and lack control groups, increasing the risk of bias. Additionally, follow-up periods are often short, making it difficult to assess long-term efficacy.

There is also a lack of consensus regarding optimal technique, including injectate type, volume, and frequency of treatment. Standardisation will be essential for future research and clinical implementation.

## 6 Limitations

- Heterogeneity in study design and methodology
- Small sample sizes in many included studies
- Predominance of low-level evidence
- Lack of high-quality comparative studies
- Limited long-term follow-up
- Potential publication bias
- Lack of standardized procedural protocols
- Concurrent treatments (e.g., corticosteroids, physiotherapy) were frequently used

## 7 Conclusion

Ultrasound-guided hydrodissection may be a safe intervention based on limited observational data. There is currently insufficient high-quality evidence to support its effectiveness for joint-related pain. It shows particular promise in adhesive capsulitis and periarticular syndromes, where mechanical factors play a significant role in symptom generation.

While early results are encouraging, the current evidence base remains limited. High-quality randomised controlled trials are needed to establish efficacy, optimise techniques, and define its role within clinical practice.

## 8 Future directions

Future research should focus on:

- Standardisation of technique and injectate protocols
- Large-scale randomised controlled trials
- Comparative studies vs. corticosteroid injection and physiotherapy
- Long-term outcome assessment
- Cost-effectiveness analyse

## Data Availability

The original contributions presented in the study are included in the article/Supplementary Material, further inquiries can be directed to the corresponding author.
