# Supplementary material for: Hydrodissection and hydrodilatation for joint pain: a scoping review
Source: Front Pain Res (Lausanne). 2026 Jul 23;7:1851291. doi: 10.3389/fpain.2026.1851291 (PMC13443037; doi:10.3389/fpain.2026.1851291)
Supplement: Supplementary file 1 [file Supplementaryfile1.docx]

Supplementary Material 1

PubMed Search Strategy

The following search strategy was used in PubMed to identify relevant studies. The search combined Medical Subject Headings (MeSH) and free-text terms related to hydrodissection and joint or periarticular pain conditions. Boolean operators and truncation were applied to maximise sensitivity.

("hydrodissection"[Title/Abstract]

OR "ultrasound-guided hydrodissection"[Title/Abstract]

OR "perineural injection"[Title/Abstract]

OR "interfascial injection"[Title/Abstract]

OR "capsular distension"[Title/Abstract])

AND

("joint pain"[Title/Abstract]

OR "musculoskeletal pain"[Title/Abstract]

OR "shoulder pain"[Title/Abstract]

OR "hip pain"[Title/Abstract]

OR "knee pain"[Title/Abstract]

OR "sacroiliac pain"[Title/Abstract])

OR

("adhesive capsulitis"[MeSH Terms]

OR "adhesive capsulitis"[Title/Abstract]

OR "frozen shoulder"[Title/Abstract]

OR "capsular fibrosis"[Title/Abstract])

OR

("osteoarthritis"[MeSH Terms]

OR "knee osteoarthritis"[Title/Abstract])

OR

("greater trochanteric pain syndrome"[Title/Abstract]

OR "trochanteric bursitis"[MeSH Terms])

AND

("ultrasound"[MeSH Terms]

OR "ultrasound-guided"[Title/Abstract]

OR "sonographic guidance"[Title/Abstract])

AND

("2010/01/01"[Date - Publication] : "2025/03/31"[Date - Publication])

AND

(English[Language])
