# Supplementary material for: Hydrodissection and hydrodilatation for joint pain: a scoping review
Source: Front Pain Res (Lausanne). 2026 Jul 23;7:1851291. doi: 10.3389/fpain.2026.1851291 (PMC13443037; doi:10.3389/fpain.2026.1851291)
Supplement: Supplementary file 2 [file Supplementaryfile2.docx]

Supplementary Material 2

## JBI Critical Appraisal of Included Case Reports

**Checklist domains (JBI Case Report Tool):**

1. Clear patient demographics
2. Clear patient history/timeline
3. Clear clinical condition on presentation
4. Diagnostic methods clearly described
5. Intervention/procedure clearly described
6. Post-intervention clinical condition described
7. Adverse events reported
8. Takeaway lessons provided

| **Study** | **1** | **2** | **3** | **4** | **5** | **6** | **7** | **8** | **Overall Appraisal** |
| --- | --- | --- | --- | --- | --- | --- | --- | --- | --- |
| Omodani 2024 | Y | Y | Y | Y | Y | Y | N | Y | Moderate |
| Sullivan 2025 | Y | N | Y | Y | Y | Y | N | Y | Moderate |
| Dosal 2023 | Y | Y | Y | Y | Y | Y | N | Y | Moderate |
| Hu 2022 | Y | Y | Y | Y | Y | Y | N | Y | Moderate |
| Omodani 2023 | Y | Y | Y | Y | Y | Y | N | Y | Moderate |
| Yoshinaga 2025 | Y | Y | Y | Y | Y | Y | N | Y | Moderate |
| Marrero Borrero 2026 | Y | Y | Y | Y | Y | Y | N | Y | Moderate |
| Naoi 2025 | Y | Y | Y | Y | Y | Y | N | Y | Moderate |
| Dougherty 2025 | Y | Y | Y | Y | Y | Y | N | Y | Moderate |

All included case reports demonstrated moderate methodological quality based on JBI criteria.

Common strengths included clear descriptions of patient presentation, diagnostic approach, and procedural technique.

<u>However, consistent limitations were identified:

- Lack of reporting of adverse events
- Incomplete reporting of patient timelines in some studies
- Absence of standardised outcome measures

Importantly, the inherent limitations of case report design (absence of controls, high susceptibility to bias, and lack of generalisability) result in an overall high risk of bias across this body of evidence.
