# Supplementary material for: Hydrodissection and hydrodilatation for joint pain: a scoping review
Source: Front Pain Res (Lausanne). 2026 Jul 23;7:1851291. doi: 10.3389/fpain.2026.1851291 (PMC13443037; doi:10.3389/fpain.2026.1851291)
Supplement: Supplementary file 3 [file Supplementaryfile3.docx]

Supplementary Material 3

Clinical Outcomes from Studies

| **Study** | **Pathology** | **Pain scale** | **Baseline pain** | **Follow-up pain** | **Improvement** | **Functional outcome** | **ROM / mobility** |
| --- | --- | --- | --- | --- | --- | --- | --- |
| Hsu 2024 | Myofascial pain syndrome (neck/shoulder) | VAS | 3.7 | 1.4 | Significant reduction | SPADI =  45 control  36 trial  NDI =  12 control  13 trial | Improved shoulder function  No Change to neck function |
| Omodani 2024 | Axillary + thoracodorsal neuropathic shoulder pain | NR | NR | NR | Symptomatic improvement | Return to sport (gymnast) | Improved shoulder movement |
| Vita 2024 | De Quervain’s tenosynovitis | VAS | 7.65 ± 1.31 | 1.65 ± 2.32 | Significant reduction | DASH score:  Pre treatment  45.55 ±2.46  2/12 post 16.34 ± 2.42  6/12 post  6.32 ± 2.37  PRWH-E  Pre treatment  55.1 ± 2.54  2/12 post  22.65 ± 4.52  6/12 post  7.45 ± 1.34 | Improved thumb/wrist (PRWH-E) & arm, shoulder and hand (DASH) |
| Sullivan 2025 | Adhesive capsulitis + OA | VAS | 8 | 3 | Pain relief reported | ASES  Pre treatment  41  2/52 Post  84  2/12 Post  78  6/12 Post  72 | Improved ROM and pain in shoulder |
| Chavez-Chiang 2012 | Rheumatoid knee arthritis | VAS | 7.6 | 2.2 | Clinical improvement | Functional outcome not recorded | N/A |
| Dosal 2023 | Chronic Achilles tendinopathy | NPRS | 3/10 at rest  9/10 breakthrough | 1/10 | Improvement in pain | Functional ankle improvement | Improved gait tolerance |
| Eliot Hu 2022 | Hunter’s canal syndrome | NR | NR | NR | Symptom resolution reported | Sport return possible | Improved knee flexion comfort |
| Skinner 2017 | Intersection syndrome wrist | NR | NR | NR | Symptom improvement | Functional wrist use improved | Improved wrist motion |
| Omodani & Takahashi 2023 | Sural neuropathy post calcaneal surgery | NR | NR | NR | Pain reduction | Improved walking tolerance | Not noted |
| Yoshinaga 2025 | ACNES-like neuropathic pain | NRS | 5/10 | 1/10 | Marked improvement | ODI  Pre Treatment  36%  1/52 post  8%  4/52 post  8%  10/52 post  6% | Reduced movement pain |
| Go 2022 | Brachial plexus injury | NR | NR | NR | Neurological recovery noted | Functional upper limb recovery | Partial ROM recovery |
| Burke 2019 | Piriformis syndrome | NR | NR | NR | Symptom improvement | Functional sitting/walking improved | Improved hip mobility |
| Chen 2025 | Myofascial pain syndrome | VAS | 5.07 | 2.32 | Significant reduction | Functional score improved | Improved regional mobility |
| Marrero 2026 | Radial nerve palsy (delayed neuropathy) | NR | NR | NR | Improvement after hydrodissection | Pre hydrodissection – No wrist extension possible  6/52 post - Medical Research Council 3/5 | Improved wrist extension |
| Rogers 2012 | Perineural catheter complication | NR | NR | NR | Procedural outcome only | Technical success | NR |
| Koonen 2023 | Lateral elbow tendinopathy | NRS | 7.5 | 1.75 | Improvement vs comparators | Functional elbow score improved | Improved grip/arm use |
| Liao 2025 | Neurogenic thoracic outlet syndrome | VAS | 7 | 2 | Significant pain reduction | DASH  Pre treatment  85  1/52 post  34  6/52 post  32.5 | Improved shoulder ROM |
| Saito 2025 | Buttock pain post hip arthroscopy | NRS | 5  7 | 1  2 | Symptom improvement | KFD pre treatment  Week 0  Pt 1 - 26.8  Pt 2 – 21  Conclusion  Pt 1 - 22  Pt 2 – 20  External Rotation Strength  Week 0  Pt 1 - 2  Pt 2 – 2  Conclusion  Pt 1 - 4  Pt 2 – 4 | Improved hip ROM |
| Naoi 2025 | Post SLAP repair shoulder pain | NRS | 7 | 3 immediately  0 long term | Pain reduction | MMT grade  Pre - 3  Post - 5 | Improved shoulder ROM |
| Lin 2020 | Carpal tunnel syndrome | VAS | Baseline = 0 | After 24/52  4ml =  -3.17  2ml =  -3.14  1ml =  -1.90 | Dose-dependent improvement | BCTQ  4ml = -15.52  2ml = -14.76  1ml = 10.62 | Improved hand function |
| Dougherty 2025 | Brachial plexus injury (iatrogenic) | NR | NR | NR | Clinical improvement | Functional recovery reported | Improved upper limb ROM |
