# Supplementary material for: Hydrodissection and hydrodilatation for joint pain: a scoping review
Source: Front Pain Res (Lausanne). 2026 Jul 23;7:1851291. doi: 10.3389/fpain.2026.1851291 (PMC13443037; doi:10.3389/fpain.2026.1851291)
Supplement: Supplementary file 4 [file Supplementaryfile4.docx]

Supplementary Material 4

Summary table detailing the injection protocols reported for the four principal pathologies identified in the literature

| **Pathology** | **Target Structure** | **Injectate Composition** | **Volume** | **Ultrasound-guided Approach** | **Pain Outcome** | **Functional Outcome** | **Mobility Outcome** |
| --- | --- | --- | --- | --- | --- | --- | --- |
| Adhesive Capsulitis | Axillary nerve, rotator interval, posterior capsule | D5W ± local anaesthetic ± corticosteroid | 20-40 mL | Anterior/posterior shoulder approach | VAS reduction average 6.3 on 10 point scale | Functional score improvement | ROM improvement |
| Greater Trochanteric Pain Syndrome | Trochanteric bursa, gluteal tendon plane | D5W ± local anaesthetic | 3-10 mL | Lateral hip approach | VAS/NRS average reduction of 5.4 on 10 point scale | Walking/ function score | Hip ROM improvement |
| Sacroiliac Joint Dysfunction | Unable to find enough information to draw a conclusion from | | | | | | |
| Femoroacetabular Impingement Syndrome | Pericapsular nerve branches/capsular plane | D5W ± local anaesthetic | 10 mL | Anterior hip approach | VAS/NRS average reduction of 4.5 on 10 point scale | Hip function score | Hip ROM improvement |
